# Developing a model for estimating infarction onset time based on computed tomography radiomics in patients with acute middle cerebral artery occlusion

**DOI:** 10.1186/s12880-021-00678-1

**Published:** 2021-10-11

**Authors:** Xuehua Wen, Zhenyu Shu, Yumei Li, Xingfei Hu, Xiangyang Gong

**Affiliations:** 1 Department of Radiology, Zhejiang Provincial People’s Hospital, Affiliated People’s Hospital, Hangzhou Medical College, Hangzhou, Zhejiang China; 2 Institute of Artificial Intelligence and Remote Imaging, Hangzhou Medical College, Hangzhou, Zhejiang China

**Keywords:** Stroke, Middle cerebral artery occlusion, Computed tomography, Radiomics, Combined model, Artificial intelligence

## Abstract

**Background:**

Radiomics analysis is a newly emerging quantitative image analysis technique. The aim of this study was to extract a radiomics signature from the computed tomography (CT) imaging to determine the infarction onset time in patients with acute middle cerebral artery occlusion (MCAO).

**Methods:**

A total of 123 patients with acute MCAO in the M1 segment (85 patients in the development cohort and 38 patients in the validation cohort) were enrolled in the present study. Clinicoradiological profiles, including head CT without contrast enhancement and computed tomographic angiography (CTA), were collected. The time from stroke onset (TFS) was classified into two subcategories: ≤ 4.5 h, and > 4.5 h. The middle cerebral artery (MCA) territory on CT images was segmented to extract and score the radiomics features associated with the TFS. In addition, the clinicoradiological factors related to the TFS were identified. Subsequently, a combined model of the radiomics signature and clinicoradiological factors was constructed to distinguish the TFS ≤ 4.5 h. Finally, we evaluated the overall performance of our constructed model in an external validation sample of ischemic stroke patients with acute MCAO in the M1 segment.

**Results:**

The area under the curve (AUC) of the radiomics signature for discriminating the TFS in the development and validation cohorts was 0.770 (95% confidence interval (CI): 0.665–0.875) and 0.792 (95% CI: 0.633–0.950), respectively. The AUC of the combined model comprised of the radiomics signature, age and ASPECTS on CT in the development and validation cohorts was 0.808 (95% CI: 0.701–0.916) and 0.833 (95% CI: 0.702–0.965), respectively. In the external validation cohort, the AUC of the radiomics signature was 0.755 (95% CI: 0.614–0.897), and the AUC of the combined model was 0.820 (95% CI: 0.712–0.928).

**Conclusions:**

The CT-based radiomics signature is a valuable tool for discriminating the TFS in patients with acute MCAO in the M1 segment, which may guide the use of thrombolysis therapy in patients with indeterminate stroke onset time.

## Introduction

Stroke represents as a common cause of death and disability worldwide [1]. Reperfusion therapies for acute ischemic stroke mainly include the use of systemic intravenous thrombolytics and mechanical thrombectomy using different stent retrievers or thromboaspiration devices [2]. The treatment with intravenous tissue plasminogen activator (tPA) remains the fastest and easiest way to initiate acute stroke reperfusion treatment, and should continue to be the first-line treatment for patients with acute ischemic stroke within 4.5 h from onset [2]. However, due to the narrow treatment time window, approximately 30% of patients do not undergo intravenous thrombolysis, since the stroke onset time is indeterminate, although the stroke may actually have occurred within the time window [3–5].

Ischemic stroke can be visible on the magnetic resonance imaging (MRI) even within 3 min after the onset of symptoms [6], and diffusion-weighted imaging (DWI) is the gold standard for evaluating the extent of ischemic stroke [7]. However, MRI has limited availability, and cannot be promptly used in many emergent cases. Computed tomography (CT) is less time-consuming than MRI. Nevertheless, the CT density changes in ischemic tissue are usually subtle, especially at the hyperacute stage. Therefore, the precise identification of infarction on CT images remains challenging [8, 9]. Hence, it is necessary to develop a new approach that can identify the subtle changes in ischemic lesions, which may facilitate the discrimination of the time from stroke onset (TFS).

Radiomics analysis has shown promise in a variety of pathologies, including cerebrovascular disease, glioma, lung cancer, hepatocellular carcinoma, and prostate cancer, in different imaging modalities [10, 11]. It is much more quantitative when compared with traditional methods, and is able to detect features that radiologists cannot recognize, such as the randomness of image intensity (entropy) or uniformity. In addition, radiomics can provide more information about the shape, size or volume, intensity, and texture of the lesion [10]. To date, the values of the radiomics signature for discriminating the TFS in acute middle cerebral artery (MCA) occlusion (MCAO) patients in the M1 segment have not been clearly outlined. In this study, the TFS was classified into two subcategories: ≤ 4.5 h, and > 4.5 h. The present study aims to extract a radiomics signature from CT images, and construct a combined model of the radiomics signature and clinicoradiological characteristics for discriminating the TFS after acute MCAO. Furthermore, the values of the radiomics signature and the combined model for discriminating the TFS were analyzed.

## Materials and methods

### Subjects

**Fig. 1:**
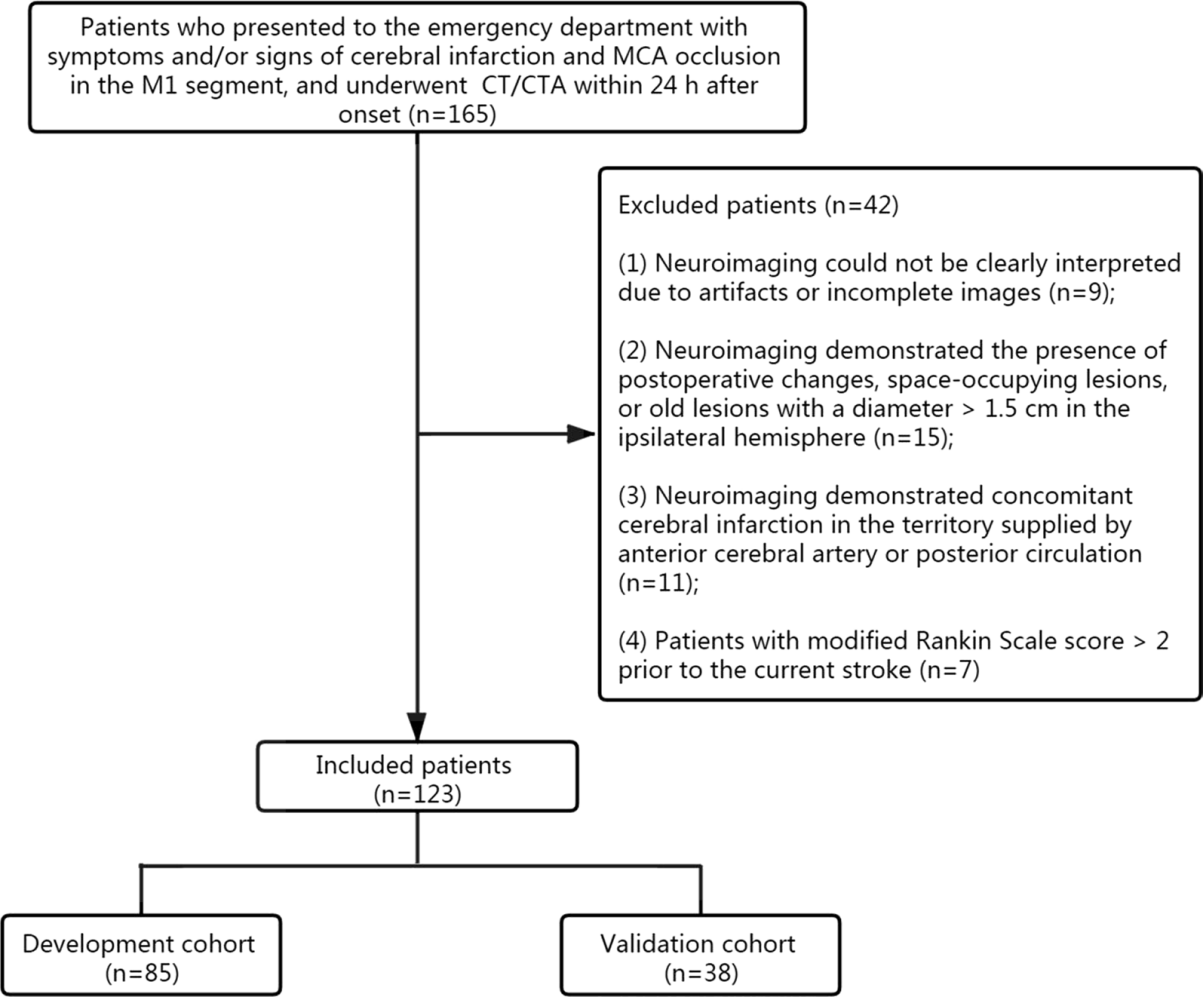
Flowchart for the subject enrollment

A total of 165 patients from Zhejiang Provincial People’s Hospital were retrieved between January 2017 and January 2021. The inclusion criteria were, as follows: (1) patients who presented with symptoms and/or signs related to ischemic stroke and with a record of stroke onset time; (2) cranial CT and CTA within 24 h after symptom onset were available; (3) a diagnosis of MCAO in the M1 segment, and ischemic infarction in the territory of the MCA was confirmed by neuroimaging. The exclusion criteria were, as follows: (1) a modified Rankin Scale (mRS) score of > 2 before admission; (2) the simultaneous presence of postoperative changes, space-occupying lesions, or old lesions with a diameter of > 1.5 cm in the ipsilateral hemisphere; (3) the neuroimaging revealed simultaneous ischemic infarction in areas supplied by the anterior cerebral artery or posterior circulation; (4) difficulty in imaging interpretation due to artifacts or incomplete images. Eventually, 123 patients were included in the present study (Fig. 1). These patients were randomly classified into two cohorts at a ratio of 7:3 [12]: development cohort (*n* = 85) and validation cohort (*n* = 38). Also, sixty eligible patients from another hospital (Tongde Hospital of Zhejiang Province) were included in our study for the external validation.

## Collection and analyses of imaging data

Two experienced neuroradiologists (raters A and B with 10- and 15-year experience in neuroradiology, respectively) reviewed and evaluated the radiological imaging, including the admission CT and CTA, as well as the follow-up images. Discordant interpretations between observers were resolved by consensus. The CT and CTA images were collected using a 640-slice CT (Toshiba, Aquilion ONE TSX-301 A). CT images were obtained with a slice thickness of 1 mm, an intersection gap of 1 mm, and a matrix of 512 × 512. CTA images were obtained with a slice thickness of 0.5 mm, an intersection gap of 0.5 mm, and a matrix of 512 × 512. The axial, sagittal and coronal CTA images were reconstructed with a slice thickness of 3 mm and an intersection gap of 2 mm.

Alberta Stroke Program Early CT Score (ASPECTS) regions (caudate nucleus, internal capsule, lentiform nucleus, insula, and 6 regions in the vascular territory of the MCA (M1–M6)) were segmented on non-contrast enhanced, reconstructed cranial CT images with 5-mm slice thickness according to a previous study [13]. Each ASPECTS area was scored 0 if abnormal and 1 if normal. Finally, these sub-scores were added to calculate the final ASPECTS for each patient (range 0 to 10).

The leptomeningeal collaterals’ status was evaluated on CTA images. Collaterals were graded according to a previous scoring system [14, 15], as follows: Grade 0, no collateral filling in the territory of the affected MCA; Grade I, collateral filling of 1–50% in the territory of the affected MCA; Grade II, collateral filling of 51–99% in the territory of the affected MCA; Grade III, 100% collateral filling in the territory of the affected MCA.

According to the CT and CTA images on admission, ASPECTS, hyperdense vessel sign (HVS) of MCA, occlusion of the intracranial internal carotid artery (ICA), and collaterals were analyzed.

## Radiomics features

**Fig. 2:**
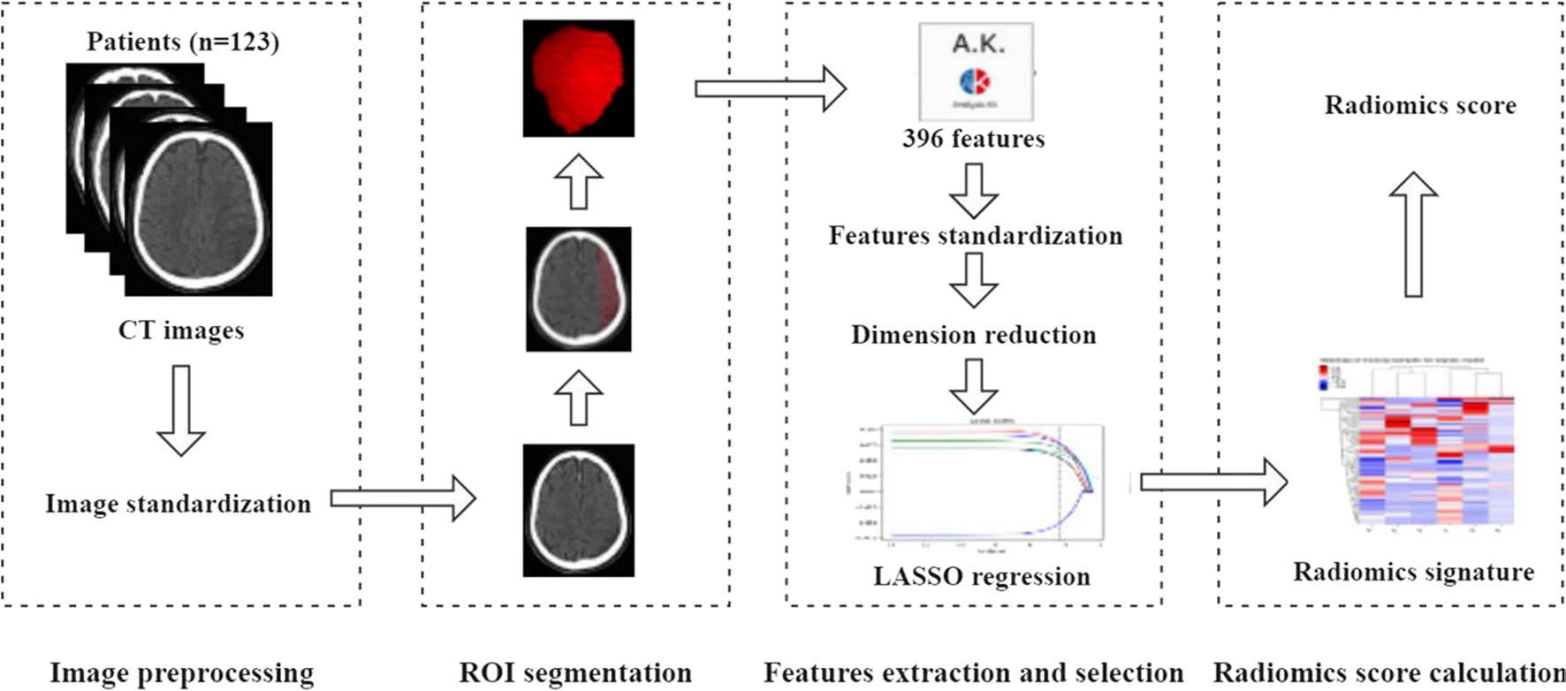
Workflow for the radiomics analysis

The radiomics analysis was performed based on the admission cranial CT, as follows (Fig. 2):

**[Pre-processing]** The pre-processing of the image data was performed using the Artificial Intelligence Kit (Version 3.0.0.R, GE Healthcare), including image interpolation, gray level discretization and intensity normalization.

**[Region-of-interest (ROI) segmentation]** Two raters (A and B) trained in neuroimaging reviewed cranial CT images of included patients. Subsequently, they annotated images from non-contrast enhanced cranial CT images by drawing polygonal ROI of the MCA territory on each slice, generating a volume of interest (VOI), using a dedicated software (ITK-SNAP software (www.itksnap.org)) according to the method of Cuocolo et al. [16]. The inter- and intra-rater agreements on the ROI segmentation were evaluated by intra-class correlation coefficients (ICCs). The inter-rater ICCs were calculated by comparing the feature extractions from raters A and B in 30 randomly selected patients. The intra-rater ICCs were calculated by comparing two measurements of rater A.

**[Feature extraction and selection]** The extracted features included histogram, formfactor, haralick, run-length matrix (RLM), gray level co-occurrence matrix (GLCM), and gray level size-zone matrix (GLSZM). A total of 396 features were extracted from each subject. These features were standardized for removing the unit limits of the data. Dimension reduction was performed using analysis of variance, Mann-Whitney *U*-test, and correlation test. The least absolute shrinkage and selection operator (LASSO) was used for further feature selection.

## Construction and validation of the radiomics signature

The radiomics signature was constructed using the multivariate logistic regression analysis, and this was used to discriminate the TFS based on the selected features after the LASSO. Then, the radiomics score (rad-score) was calculated for each subject. The calculation formula was derived from the data in the development cohort, which was used to calculate the rad-score for each subject in the validation cohort. The discriminative efficiency of the radiomics signature was evaluated using the receiver-operator characteristic (ROC) curve (AUC) in both the development and validation cohorts. The clinical efficiency of the radiomics signature for discriminating the TFS was evaluated by decision curve analysis (DCA) based on the threshold probabilities.

## Construction and validation of the combined model

All variables, including demographic characteristics, neurological functions (National Institutes of Health Stroke Scale (NIHSS) scores), risk factors (hypertension, diabetes, hyperlipidemia, etc.), and neuroimaging features (HVS, ASPECTS, collateral formation, etc.), were screened for the potential association with the TFS. Then, multivariate logistic regression analysis was used to generate the combined model for discriminating the TFS. The performance of the model was evaluated using ROC curve analyses. The efficiency of the model for discriminating the TFS was evaluated using DCA.

### Statistical analysis

SPSS 21.0 software, MedCalc 15.2.2 software, and R software (version 3.3.1) were used for the statistical analyses. The LASSO analysis was performed based on the minimum criterion by 10-fold cross validation. Mann-Whitney *U*-test, Student *t*-test, and Chi-square test were used to identify the potential variables associated with the TFS. Multivariate logistic regression analysis was used to establish the model for discriminating the TFS. The radiomics signature and combined model for discriminating the TFS were compared using the McNemar test. Also, comparison of the two ROC curves was performed using the method suggested by DeLong et al. [17]. Spearman correlation analysis was used to evaluate the correlations between the radiomics features and clinicoradiological factors associated with the TFS. A *P* value < 0.05 was considered to be statistically significant.

## Results

### Inter- and intra-rater reliability

The inter-rater agreements on the ROI segmentation between two raters ranged within 0.781–0.905.

The intra-rater agreements on the ROI segmentation between two measurements from one rater ranged within 0.797–0.928.

## Clinicoradiological characteristics

Except for the diabetes mellitus, there was no statistical difference in clinical and radiological characteristics between the development cohort and validation cohort (Table 1). There were significant statistical differences in variables (rad-score, age and ASPECTS on CT) between patients with TFS ≤ 4.5 h and patients with TFS > 4.5 h (Table 2).

**Table 1:** Characteristics of the development and validation cohorts

Variable	Development cohort (*n* = 85)	Validation cohort (*n* = 38)	*P-*value
Male gender, *n* (%)	51 (60.00%)	27 (71.05%)	0.312
Age (years), mean ± SD	72.12 ± 12.96	71.76 ± 13.07	0.889
Baseline NIHSS, median (IQR)	18 (14.50–23)	20 (13.75–27)	0.438
Hypertension, *n* (%)	56 (65.88%)	25 (65.79%)	0.992
Diabetes mellitus, *n* (%)	20 (23.53%)	3 (7.89%)	0.046
Hyperlipidemia, *n* (%)	15 (17.65%)	12 (31.58%)	0.101
Atrial fibrillation, *n* (%)	40 (47.06%)	21 (55.26%)	0.439
Smoking, *n* (%)	27 (31.76%)	15 (39.47%)	0.418
Alcohol abuse, *n* (%)	16 (18.82%)	10 (26.32%)	0.350
ASPECTS on CT, median (IQR)	7 (5–9.50)	7 (3–9)	0.520
HVS of MCA, *n* (%)	43 (50.59%)	24 (63.16%)	0.241
Collateral score, median (IQR)	1 (1–1)	1 (1–1)	0.297
Right-side MCA occlusion in the M1 segment, *n* (%)	41 (48.24%)	23 (60.53%)	0.244
ICA occlusion, *n* (%)	33 (38.82%)	18 (47.37%)	0.430

*SD* standard deviation, *NIHSS* National Institutes of Health Stroke Scale, *IQR* interquartile range, *CT* computed tomography, *ASPECTS* Alberta Stroke Program Early CT Score, *HVS* hyperdense vessel sign, *MCA* middle cerebral artery, *ICA* internal carotid artery

**Table 2:** Characteristics for patients classified according to the time from stroke onset (TFS)

Variable	TFS ≤ 4.5 h (*n* = 46)	TFS > 4.5 h (*n* = 77)	*P-*value
Male gender, *n* (%)	31 (67.39%)	47 (61.04%)	0.563
Age (years), mean ± SD	75.17 ± 12.39	70.12 ± 12.97	0.035
Baseline NIHSS, median (IQR)	19.50 (16–22.25)	19 (13–25)	0.364
Hypertension, *n* (%)	31 (67.39%)	50 (64.94%)	0.846
Diabetes mellitus, *n* (%)	9 (19.57%)	14 (18.18%)	0.850
Hyperlipidemia, *n* (%)	12 (26.09%)	15 (19.48%)	0.394
Atrial fibrillation, *n* (%)	25 (54.35%)	36 (46.75%)	0.459
Smoking, *n* (%)	15 (32.61%)	27 (35.06%)	0.846
Alcohol abuse, *n* (%)	10 (21.74%)	16 (20.78%)	0.900
ASPECTS on CT, median (IQR)	8 (5.25–9)	6 (3–9)	0.038
HVS of MCA, *n* (%)	28 (60.87%)	39 (50.65%)	0.350
Collateral score, median (IQR)	1 (1–1)	1 (1–1)	0.409
Right-side MCA occlusion in the M1 segment, *n* (%)	19 (41.30%)	45 (58.44%)	0.093
ICA occlusion, *n* (%)	19 (41.30%)	32 (41.56%)	0.978
Rad score, mean ± SD	0.54 ± 2.39	− 0.97 ± 1.35	< 0.001

*SD* standard deviation, *NIHSS* National Institutes of Health Stroke Scale, *IQR* interquartile range, *CT* computed tomography, *ASPECTS* Alberta Stroke Program Early CT Score, *HVS* hyperdense vessel sign, *MCA* middle cerebral artery, *ICA* internal carotid artery

## Development of the radiomics signature

**Fig. 3:**
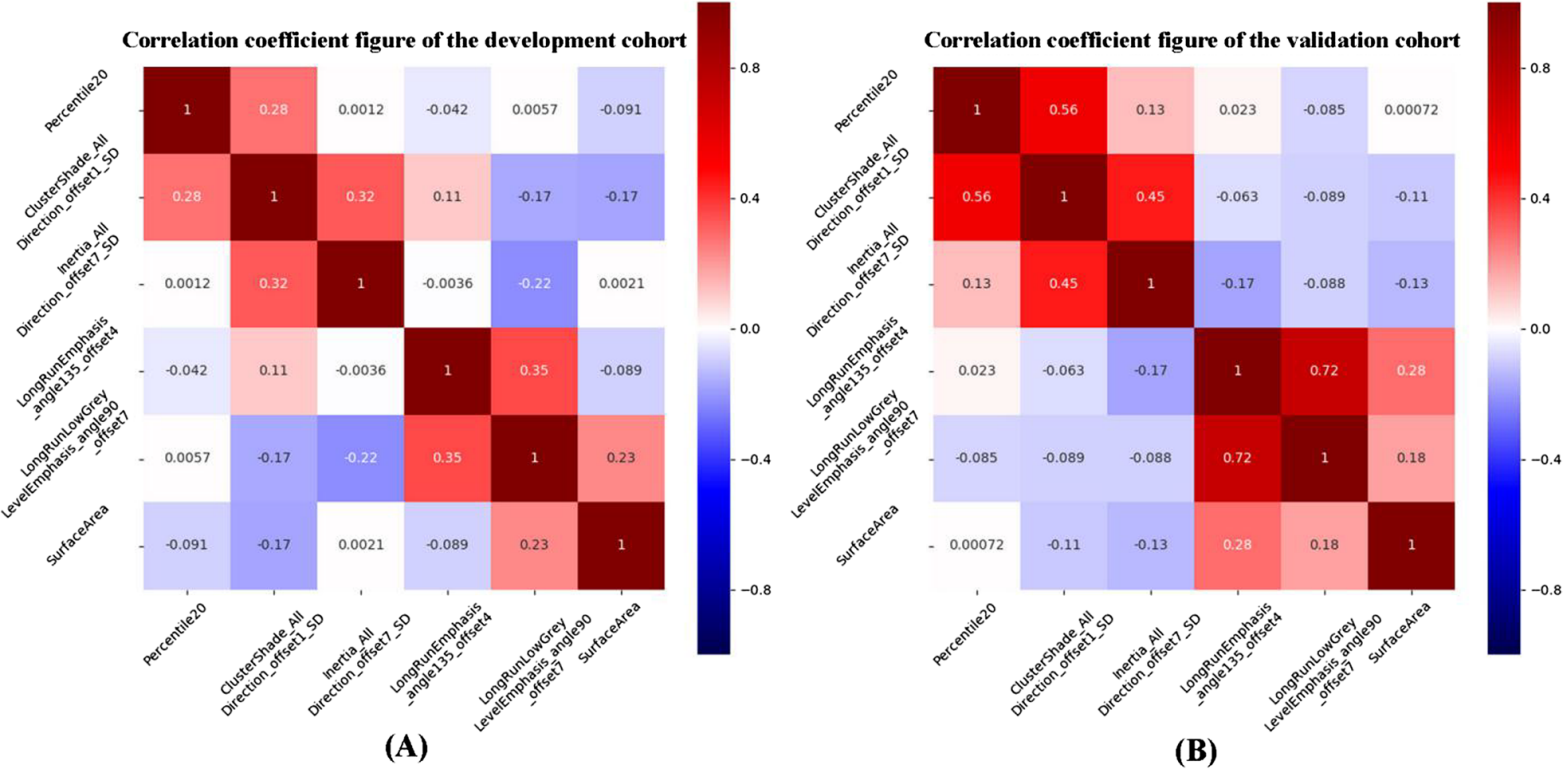
Heatmap showing the correlation between variables identified using the LASSO in the development and validation cohorts

Initially, a total of 218 features were identified by the analysis of variance and Mann-Whitney *U* test. Then, eight features were retained by the Spearman correlation analysis. The LASSO was used to further reduce the dimension (Fig. 3). Finally, the six most valuable features remained: LongRunEmphasis_angle135_offset4, SurfaceArea, Inertia_AllDirection_offset7_SD, ClusterShade_AllDirection_offset1_SD, Percentile20, and LongRunLowGreyLevelEmphasis_angle90_offset7. Subsequently, the rad-score was calculated using the LASSO model, with a linear combination of these six features. For each subject, the values of these six features were placed into the rad-score calculation formula, and the rad-score was generated to reflect the efficiency for discriminating the TFS.

**Fig. 4:**
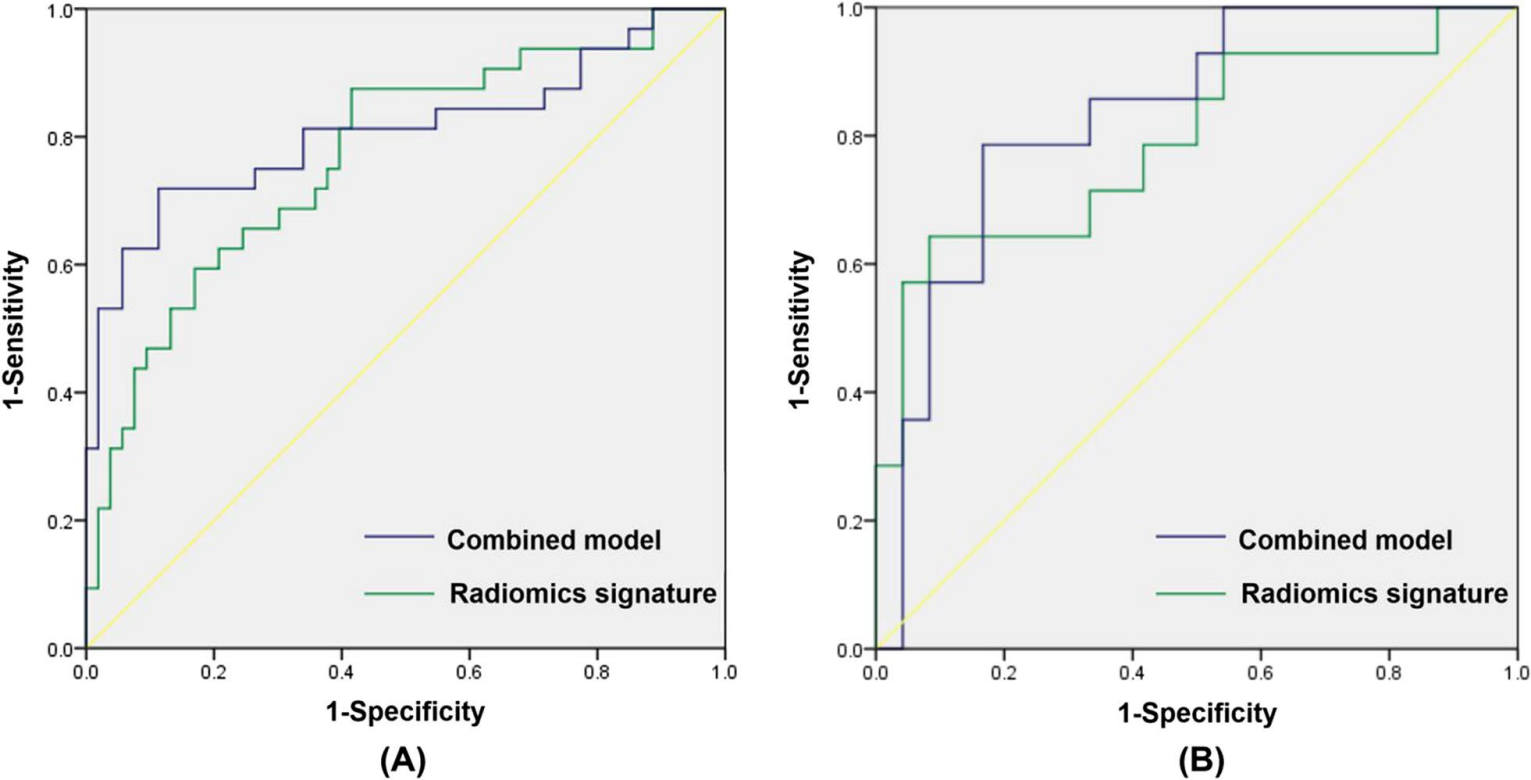
**A** The receiver operating characteristic curve for the radiomics signature (AUC, 0.770 [0.665–0.875]) and the combined model (AUC, 0.808 [0.701–0.916]) for discriminating the time from stroke onset (TFS) in the development cohort. **B** The receiver operating characteristic curve for the radiomics signature (AUC, 0.792 [0.633–0.950]) and the combined model (AUC, 0.833 [0.702–0.965]) for discriminating the TFS in the validation cohort

**Fig. 5:**
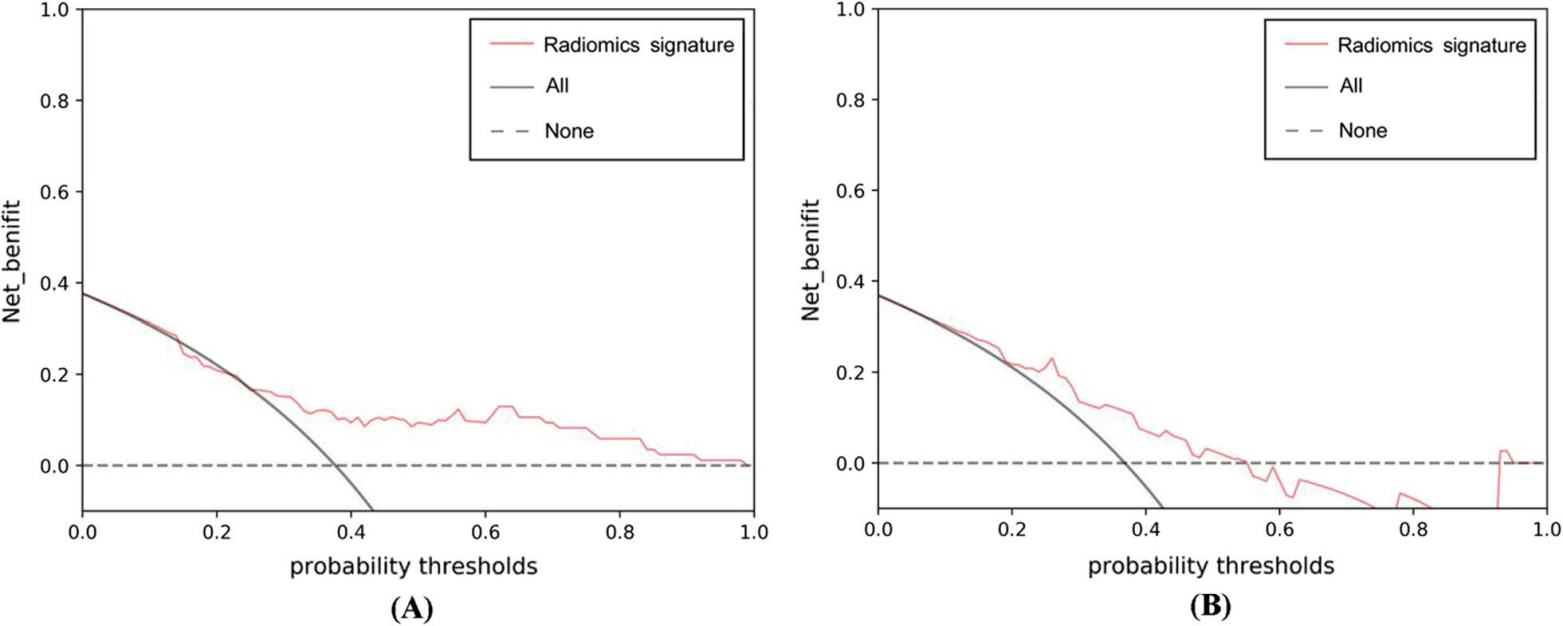
The decision curve analysis demonstrating that the radiomics signature is a valuable approach for discriminating the TFS, with a threshold probability range of 0.26–1.00 in the development cohort (**A**) and 0.10–0.55 in the validation cohort (**B**)

The AUC, sensitivity and specificity of the radiomics signature for discriminating the TFS was 0.770 (95% CI: 0.665–0.875), 87.50% and 58.49%, respectively, in the development cohort (Fig. 4). The Hosmer–Lemeshow test revealed no overfitting (*P* = 0.228). The AUC, sensitivity and specificity in the validation cohort was 0.792 (95% CI: 0.633–0.950), 64.29% and 91.67%, respectively (Fig. 4). The DCA revealed that the radiomics signature has a high power for discriminating the TFS, with threshold probabilities within 0.26–1.00 in the development cohort and threshold probabilities within 0.10–0.55 in the validation cohort (Fig. 5).

## Development of the combined model

The combined model for discriminating the TFS included the following variables: the rad-score, age, and ASPECTS on CT (Table 2).

**Fig. 6:**
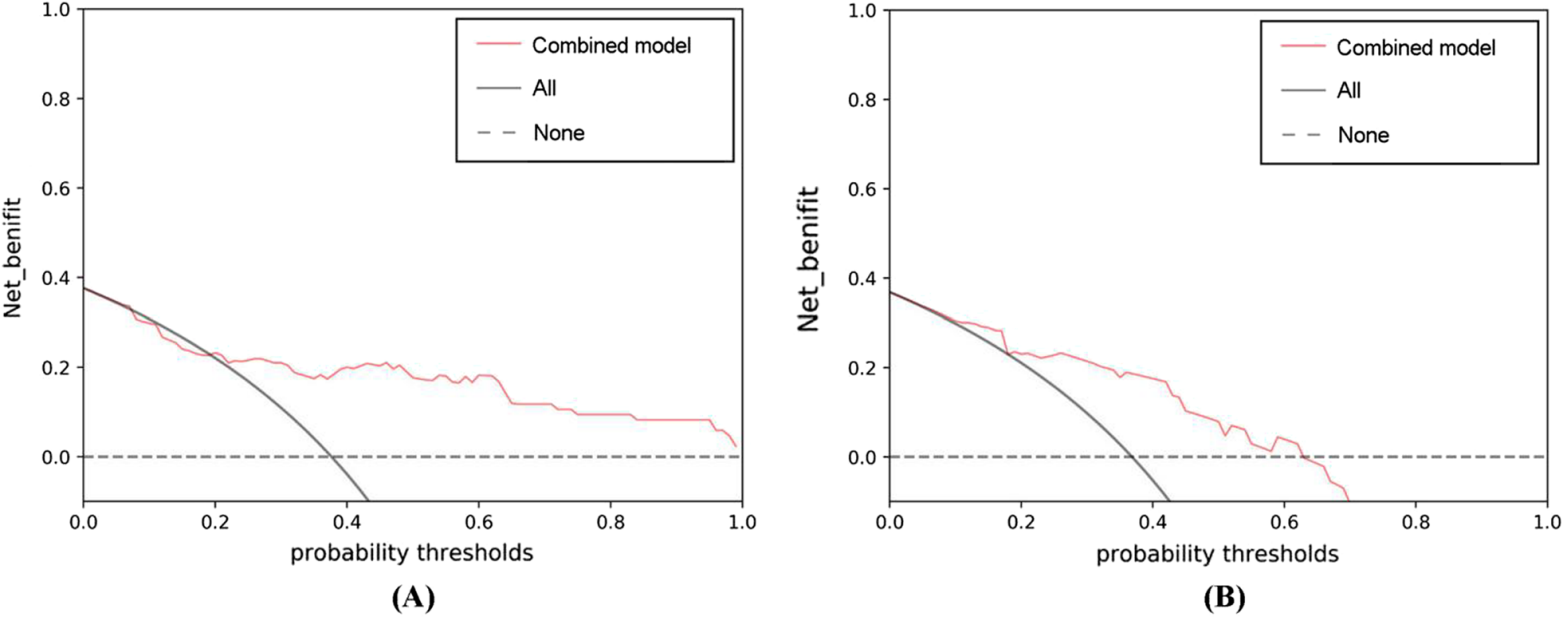
The decision curve analysis of the combined model involving the rad-score, age and ASPECTS on CT. The combined model had a better performance, with a threshold probability range of 0.19–1.00 in the development cohort (**A**) and 0.10–0.63 in the validation cohort (**B**)

The AUC, sensitivity and specificity of the combined model based on the rad-score, age and ASPECTS was 0.808 (95% CI: 0.701–0.916), 75.00% and 81.13%, respectively, in the development cohort (Fig. 4). The Hosmer–Lemeshow test revealed no overfitting (*P* = 0.124). The AUC, sensitivity and specificity in the validation cohort was 0.833 (95% CI: 0.702–0.965), 78.57% and 83.33%, respectively (Fig. 4). The DCA revealed that the combined model has a high power for discriminating the TFS, with threshold probabilities within 0.19–1.00 in the development cohort and threshold probabilities within 0.10–0.63 in the validation cohort (Fig. 6).

However, there was no statistical difference between the radiomics signature and combined model for discriminating the TFS (*P* = 0.2295). Also, in terms of the results of ROC curves, there was no statistical difference between the AUC of the radiomics signature and that of the combined model (*P* = 0.5166).

## The correlations between radiomics features and clinicoradiological characteristics

**Fig. 7:**
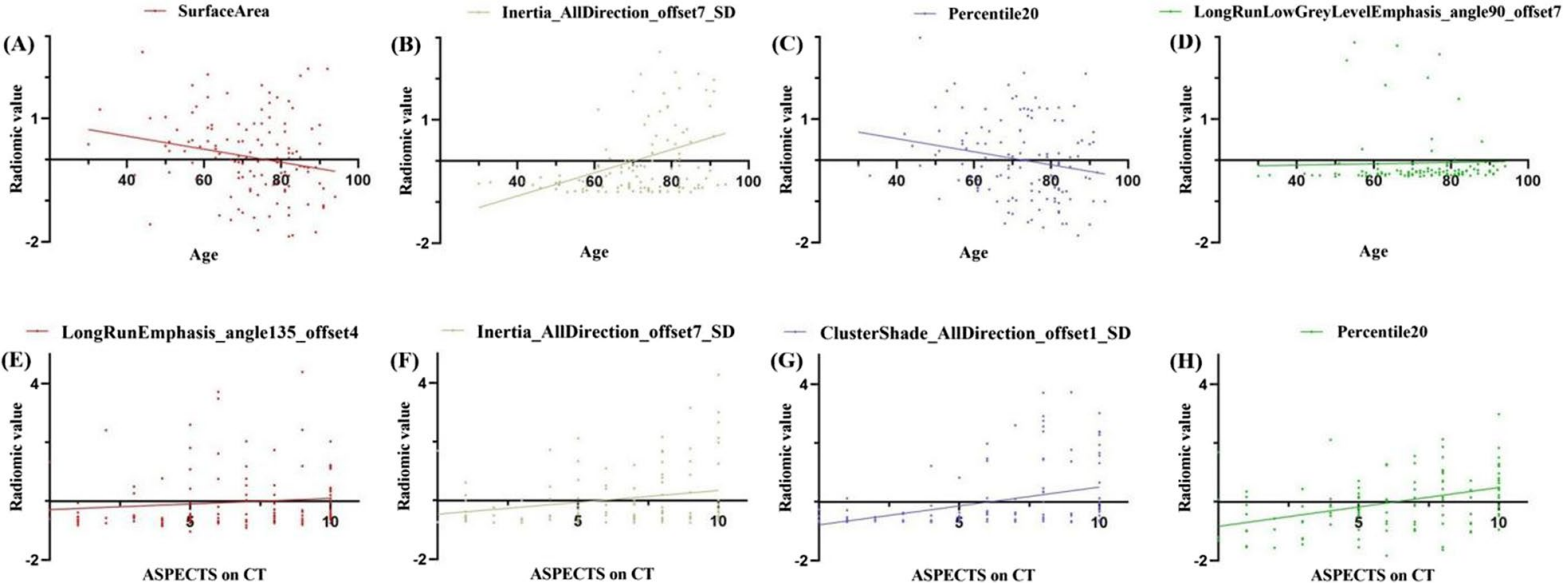
The correlation plots between radiomics features and clinicoradiological factors related to the TFS

SurfaceArea and Percentile20 were negatively correlated with age (r = − 0.218, *P* = 0.015; r = − 0.213, *P* = 0.018; Fig. 7). Inertia_AllDirection_offset7_SD and LongRunLowGreyLevelEmphasis_angle90_offset7 were positively correlated with age (r = 0.320, *P* < 0.001; r = 0.265, *P* = 0.003; Fig. 7). LongRunEmphasis_angle135_offset4, Inertia_AllDirection_offset7_SD, ClusterShade_AllDirection_offset1_SD and Percentile20 were positively correlated with ASPECTS on CT (r = 0.206, *P* = 0.022; r = 0.222, *P* = 0.013; r = 0.323, *P* < 0.001; r = 0.441, *P* < 0.001; Fig. 7).

## External validation of the radiomics signature and combined model

**Fig. 8:**
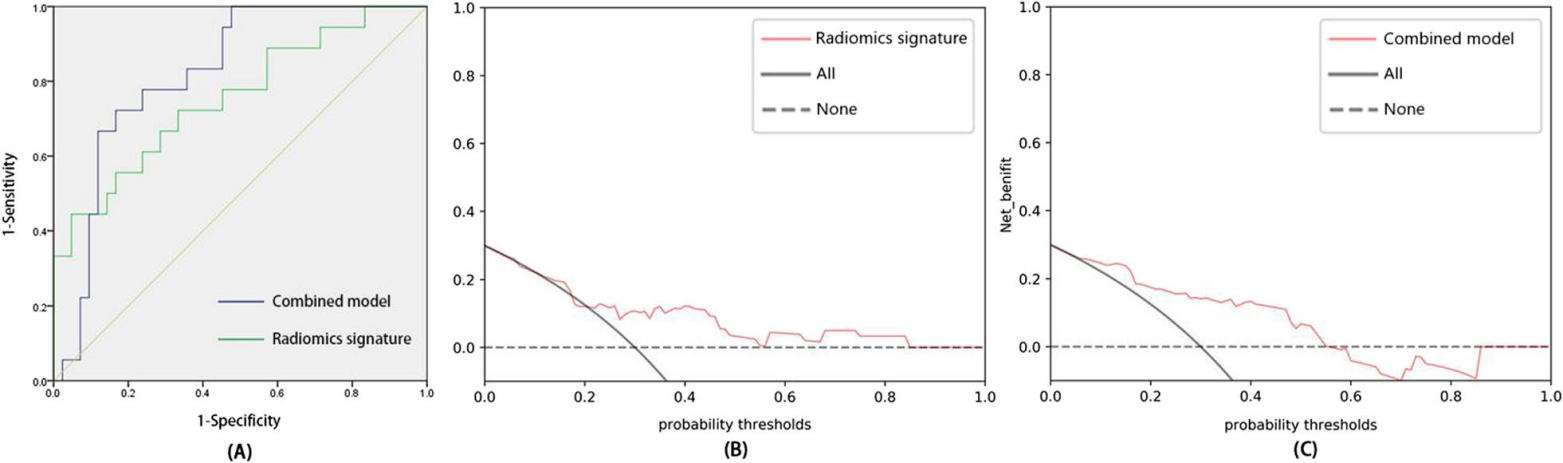
The receiver operating characteristic curve of the radiomics signature (AUC, 0.755 [0.614–0.897]) and combined model (AUC, 0.820 [0.712–0.928]) for discriminating the TFS in the external validation cohort (**A**). The radiomics signature had a a better performance with a threshold probability range of 0.20–0.86 in the external validation cohort (**B**). The combined model had a better performance with a threshold probability range of 0.06–0.57 in the external validation cohort (**C**)

The AUC, sensitivity and specificity of the radiomics signature for discriminating the TFS was 0.755 (95% CI: 0.614–0.897), 44.44% and 95.24%, respectively, in the external validation cohort (Fig. 8). The AUC, sensitivity and specificity of the combined model for discriminating the TFS was 0.820 (95% CI: 0.712–0.928), 72.22% and 83.33%, respectively, in the external validation cohort (Fig. 8). The Hosmer–Lemeshow test revealed no overfitting of the radiomics signature and combined model (*P* = 0.827; *P* = 0.541). The DCA revealed that the radiomics signature has a high power for discriminating the TFS, with threshold probabilities within 0.20–0.86 in the external validation cohort, and the combined model has a high power for discriminating the TFS, with threshold probabilities within 0.06–0.57 in the external validation cohort (Fig. 8).

## Discussion

The accurate evaluation of the TFS is pivotal for improving the clinical prognosis of ischemic stroke. Therefore, it is necessary to identify the TFS-related factors at the early stage of stroke. KERR and MARTHA [18] conducted a study that involved 336 consecutive patients with known ischemic stroke onset time, in which DWI and fluid-attenuated inversion recovery (FLAIR) imaging were performed in the emergency department. They found that the probability of stroke onset to scanning time within three hours was 66% when the FLAIR findings were negative (with or without DWI-positivity), and the FLAIR images revealed no abnormalities within six hours after stroke onset in 93% cases. In addition, MRI is not always available for stroke cases, while CT scanning is more commonly used in the emergency department. However, it is usually difficult to identify ischemic stroke lesions in the early stage on CT, although CT is convenient and has a high sensitivity for detecting intracerebral hemorrhage, which is a contraindication to thrombolytic therapy [19]. Also in the case of minor bleeding, MR T2* or gradient echo sequence may not be sufficient to distinguish acute bleeding from chronic bleeding (e.g. microbleeds), and for this, CT should be performed [19].

Radiomics analysis is a newly emerging quantitative image analysis technique. By extrapolating quantitative information from conventional images, it can identify imaging biomarkers that have important contributions to the characteristics of diseases. This method has the potential to overcome the limitations of qualitative image interpretation, which is helpful for the diagnosis, prognosis and treatment decisions [20]. Radiomics analysis has provided an alternative way for the identification [21, 22] and evaluation of cerebral infarction in recent years [11, 13, 23]. However, few studies have focused on the radiomics for discriminating the onset time of stroke. Yao et al. [24] constructed a CT-based radiomics signature for determining the onset time of symptoms in patients with basal ganglia infarction, and the radiomics signature exhibited a satisfying performance in both the development and validation cohorts. Furthermore, they also proposed that the radiomics signature may assist in therapeutic options. It is noteworthy that in the early stage of stroke, especially in the hyperacute phase, radiologists may be unable to exactly outline the extent of infarctions, and it is usually uncertain whether the basal ganglia area is involved. The MCA is the most commonly involved artery in ischemic stroke [25].

The present study evaluated the value of a CT-based radiomics signature for discriminating the TFS in patients with acute ischemic stroke (MCAO in the M1 segment). These present findings identified six features, including the RLM (LongRunLowGreyLevelEmphasis_angle90_offset7; LongRunEmphasis_angle135_offset4), texture features (Inertia_AllDirection_offset7_SD; ClusterShade_AllDirection_offset1_SD), formfactor feature (SurfaceArea), and histogram parameter (Percentile 20). The RLM, which characterizes a large area (groups of voxels) within the lesion to provide information on regional heterogeneity [26], was found to be related to the TFS. Therefore, we speculate that the lesion in ischemic stroke patients beyond 4.5 h after symptom onset might be more heterogeneous than that in patients within 4.5 h. Texture features reflect the distribution of relevant elements and the appearance of the surface, and play an important role in identifying ROI or objects in the image. Thus, they might demonstrate the surface condition (e.g. smoothness, coarseness, etc.) of the lesion caused by brain edema, or abnormal morphology of ventricles, cisterns and sulci in the pathophysiological processes of the acute cerebral infarction. Formfactor features, which represent the changes in shape, area and volume, can reflect the microscopic structures [12]. Our results indicate that the surface area of the ischemic lesion may change with the time course of the stroke. Histogram parameters can delineate the distribution of voxels in the CT imaging. The percentile, p%, of a distribution is defined as the value of the brightness a such that: P(a) = p%. Percentile 20, as one of the histogram parameters, was positively correlated with ASPECTS shown in our results. The ASPECTS is a method for assessing the extent of ischemic changes on CT. Therefore, we guess that the 20th percentile density value of the lesion on CT images in patients beyond 4.5 h after symptom onset is lower than that in patients within 4.5 h.

The present study demonstrated that the radiomics signature is associated with the clinicoradiological characteristics of stroke. In addition to the rad-score calculated from the radiomics signature, age and ASPECTS were also identified as potential factors related to the TFS. Our research indicated that the radiomics signature is a valuable tool for distinguishing the TFS ≤ 4.5 h, with an AUC of 0.770 and 0.792, in the development and validation cohorts, respectively. Interestingly, it was found that patients who presented to the emergency department for cerebral infarction within 4.5 h after symptom onset were older than those beyond 4.5 h. García-Bermejo et al. [27] also observed consistent results. In their study, patients treated for cerebral infarction with intravenous tPA within 4.5 h after symptom onset were older than those beyond 4.5 h (< 4.5 h: 72.2 ± 10.4 years old; > 4.5 h: 69.02 ± 13.2 years old). In the present study, the investigators speculated that the elderly might be more alert to the occurrence and presentation of cerebral infarction, when compared to younger individuals. Furthermore, it was found that patients who presented to the emergency department for cerebral infarction within 4.5 h after symptom onset had higher ASPECTS scores, when compared to those beyond 4.5 h. The ASPECTS score is a systematic method for assessing the extent of ischemic changes on CT [13]. The reason for this difference may be that the extent of the ischemic changes could be more easily visually perceived with the time course of the stroke.

In the present study, a model was also constructed for discriminating the TFS by combining the radiomics signature and clinicoradiological characteristics. To the best of our knowledge, the present study is the first to develop and validate a radiomics model for the TFS discrimination in acute stroke patients with MCA M1 occlusion. The combined model exhibited a relatively high efficiency, with an AUC of 0.808 and 0.833, in the development and validation cohorts, respectively, though there was no significant statistical difference between the AUC of the combined model and that of the radiomics signature. The external validation also showed the good performance of the combined model, with an AUC of 0.820 in the external validation cohort.

Limitations in the present study mainly include the following: (a) Due to the retrospective nature of this study, the quantitative analysis of cerebral CT perfusion cannot be performed. Therefore, we didn’t evaluate the value of perfusion imaging for discriminating the TFS. (b) The amount of data is relatively small. A further study on more populations is necessary to verify and possibly expand these results. (c) The median collateral score was 1 (interquartile range (IQR), 1–1) for both development and validation groups. This might be a weakness of the study because the whole population in question would have few collaterals.

## Conclusions

Our study establishes a model for discriminating the TFS in patients with acute MCAO, which may be helpful for the accurate discrimination of the onset time, and may guide the clinical decision-making.

## Data Availability

The datasets used during the current study are available from the corresponding author on reasonable request.
